# Incidence and Risk Factors of Hypogonadism in Male Patients With Latent Autoimmune Diabetes and Classic Type 2 Diabetes

**DOI:** 10.3389/fendo.2021.675525

**Published:** 2021-05-31

**Authors:** Meili Cai, Ran Cui, Peng Yang, Jingyang Gao, Xiaoyun Cheng, Chunjun Sheng, Hong Li, Hui Sheng, Shen Qu, Manna Zhang

**Affiliations:** Department of Endocrinology & Metabolism, Shanghai Tenth People’s Hospital, Tongji University School of Medicine, Shanghai, China

**Keywords:** hypogonadism, type 2 diabetes, adult latent autoimmune diabetes (LADA), low testosterone in men with diabetes, risk factors

## Abstract

**Objectives:**

This study aimed to compare the prevalence of hypogonadism between male patients with latent autoimmune diabetes (LADA) and type 2 diabetes (T2DM) and investigate the risk factors for hypogonadism in these patients.

**Methods:**

This cross-sectional study evaluated 367 male patients with LADA (n=73) and T2DM (n=294) who visited the endocrinology department of Shanghai Tenth People’s Hospital between January 2016 and October 2019 for diabetes management. Sex hormones, lipid profiles, sex hormone-binding globulin (SHBG), glycosylated hemoglobin A1c, beta-cell function, uric acid, and osteocalcin were determined in serum samples. Hypogonadism was defined as calculated free testosterone (cFT) less than 220 pmol/L along with the presence of symptoms (positive ADAM score).

**Results:**

The rate of hypogonadism in the LADA and T2DM group were 8.2, and 21.7%, respectively (p=0.017). After adjusting possible confounders, the rate of hypogonadism in the LADA group was comparable to those of the T2DM group. Univariate logistic regressions demonstrated that age, BMI, fasting C-peptide, triglycerides, total cholesterol and uric acid were associated with hypogonadism in men with diabetes, BMI, triglycerides and estradiol were independent risk for hypogonadism in men with diabetes.

**Conclusion:**

This is the first evidence to explore the rate of hypogonadism in male patients with latent autoimmune diabetes (LADA). In the population requiring admission to a large urban hospital in China, the rate of hypogonadism was comparable to those of the T2DM group after adjusting for possible confounders. BMI, triglycerides and estradiol were independently associated with the presence of HH in male diabetic patients.

## Introduction

Latent autoimmune diabetes in adults (LADA) is a highly heterogeneous disease and the most common form of adult-onset autoimmune diabetes. Patients with LADA initially do not require insulin but eventually become insulin dependent after a rapid decline in beta cell mass and function (1, 2). Many epidemiological studies have shown that the prevalence of LADA was 3.3- to 12.2-fold higher than that of adult‐onset type 1 diabetes (T1DM), with LADA accounting for 1.5% to 14% of patients previously diagnosed with type 2 diabetes (T2DM) (3). These findings suggest the important role of LADA in the wide spectrum of diabetes.

Hypogonadism is characterized by low concentrations of testosterone and is widely associated with insulin resistance, diabetes, obesity, and metabolic syndrome. Increasing evidence has shown that the high prevalence of hypogonadism in male patients with T2DM and testosterone supplement could improve glucose metabolism and insulin sensitivity in those patients (4–6). There is a bidirectional relationship between hypogonadism and diabetes. For one thing, testosterone could up‐regulate the expression of glucose transporter 4 (GLUT4) and insulin receptor substrate 1 to stimulate glucose uptake into muscle and adipose (7); for another, insulin resistance, inflammatory mediators and increased aromatase activity could reduce LH concentrations, resulting in decreased testosterone (8). Although there have been reports of hypogonadism in male patients with classical T1DM (9, 10), no study has evaluated the incidence rate of hypogonadism in male patients according to the specific types of diabetes. Previous studies reported that many clinical features in LADA are continuous spectrums between classical T1DM and T2DM (1, 2), for instance, the prevalence of metabolic syndrome in the patients with LADA fell in between classical T1DM and T2DM (11, 12); the prevalence of cardiac autonomic neuropathy and osteoporosis in LADA were comparable to T1DM but different from T2DM (13, 14). Thus, it is important to investigate the clinical characteristic of hypogonadism in patients with LADA.

Accordingly, this study aimed to compare the occurrence rate of hypogonadism among male patients with diabetes according to its specific type(LADA, and classical T2DM) and investigated the risk factors for hypogonadism in men with diabetes, which would help us to explore the association of hypogonadism and the etiology of hyperglycemia.

## Patients and Methods

### Study Design and Patients

This cross-sectional study was approved by the Institutional Human Subjects Review Board of Shanghai Tenth People’s Hospital. Written informed consent was obtained from all participants. We evaluated male patients with diabetes who visited the Endocrinology Department of Shanghai Tenth People’s Hospital between January 2016 and October 2019 for better control and management of diabetes. Patients with moderate or severe liver and renal dysfunction, with history of congenital/hypogonadotropic hypogonadism, hyperthyroidism, varicocele, epididymitis, testicle injury, abnormal karyotype, or those who received testosterone replacement therapy in the past 6 months were excluded. The cohort involved 367 patients; of them, 73 patients had LADA; and 294 patients, T2DM.

### Diagnosis and Measurements

The diagnostic criteria for LADA were glutamic acid decarboxylase (GAD) antibody-positivity, initially non–insulin requiring for at least 6 months, and diagnosed over the age of 30 years (15). Classical T2DM were diagnosed according to the criteria set by the World Health Organization. The androgen deficiency in the aging male (ADAM) questionnaire was completed by the patient with low testosterone levels (cFT<220 pmol/L). Hypogonadism was defined as calculated free testosterone (cFT) less than 220 pmol/L along with the presence of symptoms (positive ADAM score) (16, 17). It was further classified as primary hypogonadism and hypogonadotropic hypogonadism according to the levels of luteinizing hormone (LH) and follicle-stimulating hormone (FSH). Specifically, if the level of LH or FSH was less than 10 IU/L, the patient was diagnosed with hypogonadotropic hypogonadism; otherwise, the patient will be diagnosed with primary hypogonadism (18).

The participant’s medical history, body mass index (BMI), and hypertension were recorded. Blood samples were obtained from all patients in the morning after at least 10 hours of overnight fasting. Fasting plasma glucose, fasting C-peptide, glycosylated hemoglobin A1c (HbA1c), GAD antibodies, creatinine, uric acid, total calcium, total cholesterol, triglyceride, high-density lipoprotein cholesterol, and low-density lipoprotein cholesterol (LDL-c) were measured. A standard meal load test (70 g of instant noodles equivalent to an energy intake of 500 kilocalories) was performed, followed by examination of plasma glucose and C-peptide in 120 min (19). The total testosterone, estradiol, LH, FSH and SHBG were determined by the electrochemiluminescence immunoassay [(Roche Diagnostics GmbH, Cot. Germany). Testosterone: intra- & interassay CV ≤ 1.2%-18.1%, lower limit of detection: 0.025 ng/ml, measurement range: 0.025-15 ng/ml; LH: intra- & interassay CV ≤ 0.8%-1.8%, lower limit of detection: < 0.100 mIU/mL, measurement range: 0.100-200 mIU/mL; FSH, intra- & interassay CV ≤ 1.3-2.8%, lower limit of detection: 0.100 mIU/mL, measurement range: >0.100-200 mIU/mL]. Serum osteocalcin (OC) was tested using an N-MID osteocalcin enzyme-linked immunosorbent assay kit (Elecsys, Roche diagnostic Ltd., Switzerland). Sartorius et al.’s formula (cFT=24.00314xT/log10(SHBG)-0.04599xT^2^) was adopted to calculate free testosterone concentrations (20, 21).

The microvascular disease was defined as presence of macroalbuminuria, diabetic neuropathy and diabetic retinopathy. The macrovascular disease was defined as presence of cerebrovascular disease, coronary heart disease, heart failure, or peripheral arterial disease. Plaques were identified by the ultrasonography test, including the common carotid artery, superficial carotid artery, common femoral artery and superficial femoral artery. The above data were collected by reviewing of all patient’s electronic medical records.

### Statistical Analysis

Continuous data were compared among the two groups using linear regression analyses. Meanwhile, categorical data were compared among the three groups using logistic regression analyses. Multiple logistic regression models were used to determine the effect of diabetes types on the diagnosis of hypogonadism and investigate the independent risk factors of hypogonadism. Variables with P<0.20 in univariate analysis were included in the multiple logistic regression analyses. All statistical analyses were performed using SPSS version 20.0 software. A P value of <0.05 was considered statistically significant.

## Results

The mean age of the patients with LADA and T2DM was 53.6 ± 13.2 and 51.9 ± 16.3 years, respectively. Considering the BMI was significantly different in two groups, the comparison of clinical features between them was adjusted by BMI. The LADA group had lower SBP than the T2DM group. The mean fasting c-peptide, postprandial c-peptide, uric acid and triglyceride levels in the LADA group were lower than those of the T2DM group. Meanwhile, the HDL-c levels in the LADA group were higher than those of the T2DM group. The mean DBP, total cholesterol, LDL-c, creatinine and osteocalcin levels were similar between the two groups. The frequencies of microvascular disease, macrovascular disease and plaques were comparable between the two groups **(** **Table 1** **)**. There were no significant differences in LH, FSH and cFT levels between the two groups. The mean testosterone, E_2_ and SHBG levels of the LADA group were different from those of the T2DM group.

**Table 1:** General characteristics of male patients with LADA and T2DM.

Items	LADA(N=73)	T2DM(N=294)	P Values
Age (years)	53.6 ± 13.2	51.9 ± 16.3	0.930
Age at diagnosis (years)	43.0 ± 11.7	42.9 ± 13.5	0.823
Duration of diabetes (years)	10.0 ± 8.5	10.4 ± 7.4	0.150
Family history of diabetes (%,n)	50.7 (37)	46.4 (136)	0.880
Smoking (%,n)	54.8 (40)	36.5 (107)	**0.023**
BMI (kg/m^2^)	20.8 ± 2.2	25.9 ± 4.1	**<0.001**
SBP (mmHg)	126.4 ± 19.6	136.1 ± 18.2	**0.047**
DBP (mmHg)	73.2 ± 10.6	78.4 ± 12.3	0.098
HbA1c (%)	10.4 ± 2.3	9.3 ± 2.3	0.599
Fasting serum C-peptide(ng/mL)	0.2 ± 0.2	2.1 ± 1.3	**<0.001**
Postprandial C-peptide (ng/mL)	0.4 ± 0.4	4.8 ± 3.1	**<0.001**
Serum creatinine (umol/L)	68.1 ± 15.5	71.6 ± 49.1	0.166
Uric acid (umol/L)	263.6 ± 95.7	352.9 ± 113.7	**0.003**
Total cholesterol (mmol/L)	4.4 ± 1.7	4.6 ± 1.4	0.239
Triglycerides (mmol/L)	0.8 (0.6-1.3)	1.5 (1.0-2.3)	**0.016**
HDL-c (mmol/L)	1.3 ± 0.3	1.0 ± 0.3	**0.003**
LDL-c (mmol/L)	2.5 ± 1.5	2.7 ± 1.0	0.205
Osteocalcin (ng/ml)	12.1 ± 5.1	12.8 ± 6.1	0.270
Microvascular disease (%)	41.1 (30)	47.6 (139)	0.327
Macrovascular disease (%)	20.5 (15)	28.6 (84)	0.315
Plaques (%)	53.4 (39)	48.3 (142)	0.815
LH (IU/L)	7.6 ± 4.2	7.6 ± 4.8	0.601
FSH (IU/L)	8.6 ± 6.1	9.4 ± 6.3	0.532
E_2_ (pmol/L)	104.6 ± 53.8	108.2 ± 48.5	**0.004**
Testosterone (ng/ml)	6.1 ± 2.2	4.0 ± 1.9	**0.001**
SHBG (nmol/L)	62.6 ± 15.9	34.9 ± 23.2	**0.028**
cFT (nmol/ml)	263.0 ± 134.6	204.8 ± 94.7	0.340

BMI, body Mass Index; SBP, systolic blood pressure; DBP, diastolic blood pressure; HbA1c, glycosylated hemoglobin A1c; HDL-c, high-density lipoprotein cholesterol; LDL-c, low-density lipoprotein cholesterol; LH, luteinizing hormone; FSH, follicle-stimulating hormone; E_2_, Estradiol; SHBG, sex hormone-binding globulin cFT; calculated free testosterone. All data were adjusting for BMI. The bold values indicates statistical significance.

**Figure 1:**
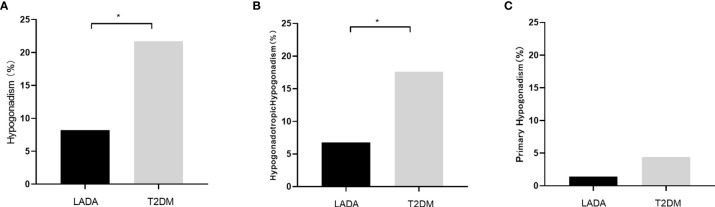
**(A)** The incidence rates of hypogonadism in the LADA and T2DM groups (8.2% *vs* 21.7%, P=0.017); **(B)** The incidence rates of hypogonadotropic hypogonadism in the LADA and T2DM groups (6.8% vs 17.6%, P=0.019); **(C)** The incidence rates of primary hypogonadism in the LADA and T2DM groups (1.4% *vs* 4.1%, P=0.318). The symbol “*” indicates statistical significance.

The rate of hypogonadism was much lower in the LADA group than the T2DM group (8.2% vs 21.7%, P=0.017). The incidence rates of hypogonadotropic hypogonadism in the LADA and T2DM groups were 6.8%, 17.6%, respectively (P=0.019). Meanwhile, the incidence rates of primary hypogonadism in the LADA and T2DM groups was 1.4% and 4.1%, respectively (P=0.318) **(** **Figure 1** **)**. To determine the influence of diabetes types along with confounders on the diagnosis of hypogonadism, multiple logistic models were used to compare the rate of hypogonadism between two groups. After adjusting possible confounders (Model 3 and Model 4), the rate of hypogonadism in the LADA group was comparable to those of the T2DM group **(** **Table 2** **)**.

**Table 2:** Multiple logistic regression of hypogonadism and type of diabetes.

Rate of hypogonadism	Reference(LADA)	Type 2 Diabetes			
		OR	95%CI		P Values
Model 1	1	**3.061**	**1.172-7.994**		**0.022**
Model 2	1	**2.942**	**1.115-7.759**		**0.029**
Model 3	1	2.289	0.738-7.093		0.151
Model 4	1	1.932	0.690-5.406		0.209

OR, odds ratio CI; confidence interval; Model 1 was unadjusted; Model 2 was adjusted for age, duration of diabetes and smoking; Model 3 was adjusted for age, duration of diabetes, smoking, family history, BMI, hypertension; Model 4 was adjusted for age, duration of diabetes, smoking, family history, BMI, hypertension, HbA1c, creatinine, triglycerides. The bold values indicates statistical significance.

The risk factors for hypogonadism in male diabetic patients was explored in the overall population. Univariate logistic regressions demonstrated that age, BMI, fasting C-peptide, triglycerides, total cholesterol and uric acid were associated with hypogonadism in men with diabetes **(** **Table 3** **)**. Moreover, BMI (OR 1.149 [95% CI: 1.033–1.279]), triglycerides (OR 1.167 [95% CI:1.014–1.343]) and estradiol (OR 0.96 [95% CI:0.976–0.995]) were independently associated with the presence of hypogonadism in the multiple logistic regression analyses. There were no association between hypogonadism status and HbA1c, fasting glucose, osteocalcin, microvascular or macrovascular complications of diabetes, and presence of plaques **(** **Table 4** **)**.

**Table 3:** Univariate logistic regressions between variables and hypogonadism status.

Variable	OR	95% CI	P Values
Age	**1.127**	**1.057-1.324**	**0.016**
BMI	**1.119**	**1.116-1.229**	**<0.001**
Fasting Glucose	1.026	0.957-1.102	0.461
Fasting C-peptide	**1.397**	**1.147-1.704**	**0.001**
HbA1c	1.004	0.890-1.131	0.952
Triglycerides	**1.242**	**1.084-1.423**	**0.002**
Total cholesterol	**1.223**	**1.010-1.479**	**0.039**
HDL-c	0.649	0.281-1.499	0.311
LDL-c	0.921	0.686-1.236	0.582
Uric acid	**1.003**	**1.002-1.005**	**0.020**
Osteocalcin	0.945	0.889-1.004	0.066
E_2_	0.995	0.989-1.001	0.128
Microvascular disease	1.173	0.593-2.215	0.532
Macrovascular disease	1.241	0.621-2.479	0.441
Plaques	0.772	0.397-1.312	0.286

OR, odds ratio CI; confidence interval; BMI, body Mass Index; HbA1c, glycosylated hemoglobin A1c; HDL-c, high-density lipoprotein cholesterol; LDL-c, low-density lipoprotein cholesterol; E_2_, Estradiol; SHBG, sex hormone binding globulin. The bold values indicates statistical significance.

**Table 4:** An adjusted Logistic regression model between variables and hypogonadism status.

Variable	OR	95% CI	P Values
Age	1.008	0.985-1.033	0.520
BMI	**1.149**	**1.033-1.279**	**0.011**
Fasting C-peptide	1.009	0.738-1.379	0.957
Triglycerides	1.167	1.014-1.343	0.031
Uric acid	1.003	0.999-1.006	0.069
Osteocalcin	0.965	0.899-1.036	0.321
**E_2_**	**0.986**	**0.977-0.996**	**0.002**

OR, odds ratio CI; confidence interval; BMI, Body Mass Index; E_2_, Estradiol. The bold values indicates statistical significance.

## Discussion

Increasing evidence has shown that hypogonadism was associated with insulin resistance, diabetes, obesity, and metabolic syndrome (4, 6). Moreover, the rate of hypogonadism is higher in patients with T2DM but lower in patients with classical T1DM (5, 9, 10). However, studies on the rate of hypogonadism in patients with LADA are limited. Given that the clinical characteristics of LADA overlaps both T1DM and T2DM, it is important to investigate testosterone levels in patients with LADA. The study would help us to explore the association of hypogonadism and the etiology of hyperglycemia.

In our study, the mean total testosterone levels in patients with LADA were significantly different from those in patients with T2DM. This was consistent with previous studies that testosterone levels tended to be normal in patients with T1DM but lower in patients with T2DM (9, 10). Given the much larger BMI of the men in the T2DM group, a low free T along with positive ADAM questionnaire were using as the threshold to “diagnose” hypogonadism in the present study. The rates of hypogonadism in patients with T2DM was 21.7%, which were generally in line with previous studies showing that hypogonadism was prevalent in 25%-40% of patients with T2DM (22, 23). It is interesting to note that compared with T2DM, patients with LADA had higher SHBG level but lower rate of hypogonadism. Previous studies have shown that obesity could aggravate low-grade inflammation, resulting in low levels of SHBG and testosterone (24, 25). Considering BMI was markedly low in the LADA group, our results might be explained by the fact that the effects of obesity on testosterone are more substantial than the effects of SHBG on testosterone in diabetic men. After controlling for confounders in multiple logistic regression models, the discrepancy between the LADA and T2DM groups was disappeared. This result indicates that the association of hypogonadism status and the etiology of hyperglycemia was limited. This result needs to be validated in studies with large sample sizes.

To explore the possible underlying mechanism for the phenomena, the risk factors for hypogonadism in male diabetic patients were explored in the overall population. Consistent with previous studies, majority of cases of hypogonadism in the two groups were hypogonadotropic hypogonadism (23, 26). This finding suggest that an inadequate release of gonadotropin is the main reason for low testosterone in diabetes patients. Our data showed that BMI was a pivotal risk factor for hypogonadism, consistent with previous studies reporting that male obesity is often accompanied with low testosterone and that BMI was negatively correlated to low testosterone in both T1DM and T2DM patients (4, 21, 26). Low testosterone levels could aggravate fat depot, especially abdominal visceral fat, which is associated with the effects of testosterone on lipid storage, lipolysis and adipogenesis (27). On the contrary, increased visceral fat results in accumulation of proinflammatory cytokines and free fatty acids that aggravate insulin resistance and dysregulated leptin signaling could inhibit hypothalamic–pituitary–gonadal (HPG) axis function (28, 29).

It is widely reported that the high expression of aromatase in the adipose tissue, which could increase the conversion of testosterone into estradiol, leading to decreased gonadotropin secretion (30). Our data have shown that estradiol was negatively associated with hypogonadism status in male diabetic patients after adjusting confounding factors. The findings were supported by a recent study reporting that lower aromatase expression in the adipose tissue of obese men were with low testosterone compared to controls with normal circulating testosterone (31). Moreover, our results further support the notion that although estradiol is important for this negative feedback, androgen receptor-mediated effects also play a role (32).

We also observed an association between uric acid and hypogonadism in our study consistent with the findings of other studies that uric acid was negatively correlated with testosterone levels in patients with T2DM (22, 23). A previous study also reported decreased mean testosterone levels in patients with gouty kidney disease and gouty arthritis (33). It was reported that the relationship between uric acid and testosterone levels were bidirectional. On one hand, high levels of uric acid could form crystals in the testicular tissue leading to oxidative damage (34). On the other hand, low levels of testosterone decrease protein synthesis, which results in accumulation of endogenous purine causing hyperuricemia (35). However, after adjusting confounder factors, there was no association between uric acid and hypogonadism. Further studies are needed to clarify whether uric acid influences gonadotropin secretion.

In addition, triglycerides and total cholesterol were risk factors for hypogonadism in male diabetic patient. The results support the findings that men with hypogonadism may have dyslipidemia and testosterone treatment could significantly decrease total cholesterol and triglycerides (6, 30). Previous studies have reported that diabetic neuropathy was associated with the levels of testosterone in patients with T2DM (36). However, there were no association between hypogonadism status and microvascular or macrovascular complications of diabetes, and presence of plaques. In line with previous studies, fasting glucose, HbA1c levels and osteocalcin were not associated with hypogonadism in the present study (37–39).

This study has some limitations. First, the exclusion of patients with CKD may be a selection bias for this study; Second, the recruited patients were all inpatients with poor blood glucose or metabolic control, which might increase the risk of hypogonadism; Third, few men without diabetes as a proper comparison may bring about a confounding bias; Fourth, the sample size of patient with LADA was not adequate to detect significant differences. Despite these limitations, we believe that our study is valuable because our findings might not only contribute to further understand the effects of testosterone on glucose metabolism, but also help to better understand the effective way to prevention hypogonadism in male patients with diabetes.

## Conclusion

In a population of requiring admission to a large urban hospital in China, the rate of hypogonadism was comparable to those of the T2DM group after adjusting confounding factors. The results suggested the association between hypogonadism and the etiology of hyperglycemia was limited in this study. BMI, triglycerides and estradiol were independently associated with the presence of HH in male diabetic patients.

## Data Availability Statement

The raw data supporting the conclusions of this article will be made available by the authors, without undue reservation.

## Ethics Statement

The studies involving human participants were reviewed and approved by The Institutional Human Subjects Review Board of Shanghai Tenth People’s Hospital. The patients/participants provided their written informed consent to participate in this study.

## Author Contributions

MZ: designed and wrote. MC, RC, and PY: performed and collected. JG, XC, and CS: participated in recruiting the patients. HS and SQ: edited. All authors contributed to the article and approved the submitted version.

## Funding

This study is supported by grants from the National Key R&D Program of China (No.2018YFC1314100) and the National Nature Science Foundation (No.81601269).

## Conflict of Interest

The authors declare that the research was conducted in the absence of any commercial or financial relationships that could be construed as a potential conflict of interest.
